# Multiple Wasp Stings Leading to Rhabdomyolysis Induced Acute Kidney Injury with Incidental Ectopic Kidney: A Case Report

**DOI:** 10.31729/jnma.7866

**Published:** 2022-10-31

**Authors:** Angel Dongol, Abhin Sapkota, Rahul Devkota, Asim Pandey, Tulsi Ram Bhattarai

**Affiliations:** 1 Department of Nephrology, Kathmandu Medical College and Teaching Hospital, Sinamangal, Kathmandu, Nepal; 2 Chirayu National Hospital and Medical Institute Pvt. Ltd, Basundhara, Kathmandu, Nepal

**Keywords:** *acute kidney injury*, *case reports*, *creatine kinase*, *hymenoptera*, *rhabdomyolysis*

## Abstract

Rhabdomyolysis refers to skeletal muscle breakdown causing a release of different intracellular proteins including myoglobin and several electrolytes in the bloodstream. Elevations diagnose rhabdomyolysis in serum creatine kinase. Mass envenomation by multiple wasp stings can cause rhabdomyolysis followed by acute kidney injury, although it is scarce. A 24-year-old male presented to our tertiary centre in an anaphylaxis-like state after multiple wasps sting, rapidly developing rhabdomyolysis followed by acute kidney injury. Despite having an ectopic kidney with a preexisting renal parenchymal disease, he recovered and was discharged, which in itself is a rare entity of low clinical incidence. Wasp stings can potentially result in serious clinical manifestations, which need to be watched over, assessed and promptly treated.

## INTRODUCTION

Multiple wasps stings can cause complications ranging from mild local reactions to life-threatening anaphylaxis which has been significantly underestimated.^1,2^ Envenomation occurs as accidents or occupational exposure, especially in tropical rural areas of Nepal.^3,4^ Rhabdomyolysis-induced AKI may be relatively benign but late diagnosis, delayed treatment and underlying comorbidities may prove fatal.^5,6^ Such occurrence in a patient with an ectopic kidney has not been reported before. We report a case of a 24-year-old male who presented after multiple wasp stings.

## CASE REPORT

**Figure 1:**
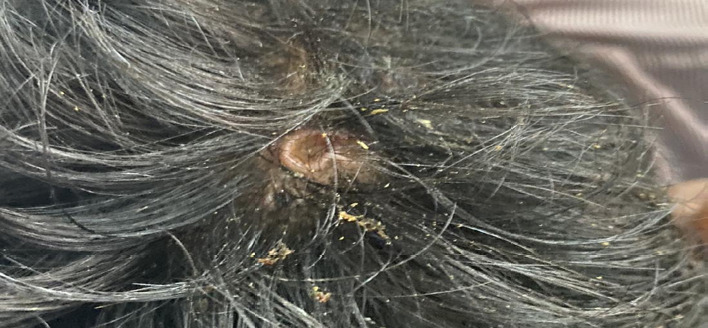
Wasp sting lesion on the patient's scalp.

A 24-year-old male presented to our emergency department 10 hours after being stung multiple times by wasps over the bilateral upper limbs, scalp and face (Figure 1).

He had accidentally disturbed a wasp hive while cutting down tree branches in a forest near his village in the hilly district. Initially, he developed severe itching all over his body along with severe headache, which was later followed by nausea, and vomiting with subsequent development of pedal oedema, facial puffiness and difficulty in breathing after which he was brought to our hospital. There was no history of trauma, exertion or any known comorbidities as per the patient.

On initial evaluation, he had an increased respiratory rate of 25/minute and blood pressure of 90/50 mmHg. Intramuscular epinephrine 0.5 mg (0.5 ml of 1 mg/ml, 1:1000) single dose was immediately administered after which some of his symptoms improved but he still had a fever, and his urine output was low. Lab investigations showed leukocytosis (the total count was 13,000/mm^3^ with neutrophil as 80%) and a high blood urea nitrogen (BUN): 192 mg/dl and creatinine: 4.1 mg/dl. Routine examination of urine shows albumin to be 3+ with packed red blood cells 6-8/hpf and granular casts 5-7/hpf. Spot urine protein: creatinine ratio was also high indicating proteinuria. Creatine kinase (CK) was 4950 U/l (range 26-308 U/l) which is indicative of rhabdomyolysis, this was accompanied by elevated cardiac bio-marker CK-MB and impaired liver function test.

The patient was admitted into the intensive care unit for further management. The rapid expansion of intravascular volume with intravenous (IV) fluid and forced alkaline diuresis with sodium bicarbonate and mannitol was done for rhabdomyolysis. His blood pressure was at 90/60 mmHg and he developed abdominal distension. His urine output failed to improve even after IV fluids loading. Repeat total counts and renal function tests two days later showed even higher leukocyte counts of 33,000/mm^3^ with BUN: 208 mg/dl and creatinine 8.2 mg/dl.

His renal function test (RFT) declined progressively in the first week of admission. His BUN was 102 mg/dl and creatinine was 3 mg/dl. His total leukocyte count dropped down to 12,000 per cumm. However, he gradually started to lose orientation. Repeated blood gas analysis showed metabolic acidosis and he was put under mechanical ventilation to prevent respiratory fatigue due to high respiratory rate from pleural effusion and acidosis.

**Figure 2:**
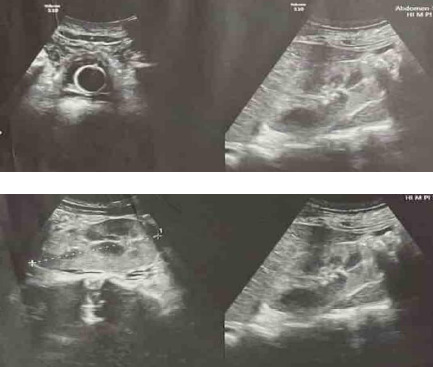
USG of the abdomen and pelvis showing right ectopic kidney.

An ultrasonography (USG) of the abdomen and pelvis was performed to evaluate his abdominal distension which showed a right normal-sized kidney with increased echotexture with loss of corticomedullary differentiation (Figure 2).

Due to his stay in the hospital, hospital-acquired pneumonia was also suspected, which was ruled out because of clear bilateral equal air entry on chest examination and a normal chest x-ray. Blood culture was sent, which returned negative and helped rule out possible bloodstream infection. Vague clinical features and inconsistent lab results without much improvement in treatment proved to be challenging.

**Figure 3:**
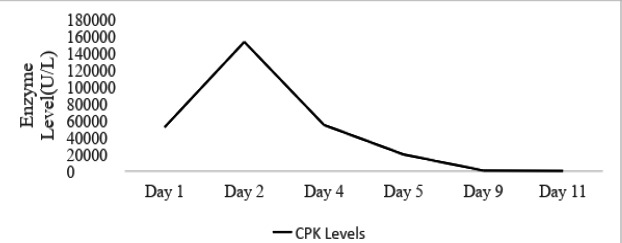
The trend of CK values since the day of the sting.

He was started on haemodialysis immediately. He started improving gradually and was extubated on the fifth day. With progressive days the levels of CK gradually returned to normal, indicating resolution of rhabdomyolysis (Figure 3).

One week after starting haemodialysis, his pleural effusion had resolved and he progressed into the polyuric phase by the end of the third week. He underwent a total of 15 sessions of haemodialysis until his RFT stabilised at serum creatinine of 2 mg/dl and subsequently, he was discharged after 32 days of hospital stay.

On follow-up, at 2 months and 6 months, his urine output was normalised and his serum creatinine was 1.0 mg/dl. There were no adverse or unanticipated events on the follow-up examination.

## DISCUSSION

The toxic compounds in the wasp venom include enzymes which increase vascular permeability, initiate inflammation and cause hemolysis.^7,8^ Locally around the site of the sting, pain and swelling are experienced. At the same time, system-wide allergic reactions may be mild, moderate (angioedema, asthma, abdominal pain) or even severe (laryngeal oedema, hypotension, loss of consciousness).^1^ Many different complications can arise such as myocardial infarction, arrhythmias, myocarditis, hepatic centrilobular necrosis, Guillain-Barre syndrome, stroke, vasculitis and haematological complications just to name a few.^8,9^

Renal complications such as AKI, nephrotic syndrome and renal tubular acidosis can also occur. Early deaths are mostly because of severe anaphylaxis and cardiac events while fatalities occurring later are the effects of renal failure.^10^ In our case as well the patient had features suggestive of rhabdomyolysis with very high CK values. This may have led to subsequent AKI as shown by his acutely raised creatinine values and high serum urea levels.

The management of massive wasp stings remains supportive with no specific anti-venom being available. Anaphylaxis must be treated promptly with epinephrine as was done in our case and repeated in 10-15 minutes if clinical improvement is not apparent. The presence of other symptoms of anaphylaxis requires additional management including airway management, fluids, vasopressors and bronchodilators based on the severity of symptoms. A short course of oral corticosteroid is often used to prevent late-phase allergic reactions. Management of the rapidly developing AKI requires prompt renal replacement therapy with either peritoneal dialysis or hemodialysis.^11^

The incidental finding of an ectopic kidney and the loss of corticomedullary differentiation in our case made the outcome unpredictable despite the patient's young age. Early diagnosis and management were crucial, which included removal of the stings and early correction of hypotension with IV fluids to maintain urine output. Forced alkaline diuresis with sodium bicarbonate and mannitol was done to flush the blocked tubules from rhabdomyolysis and hemolysis.

Although the rise in Aspartate Aminotransferase (AST) and Alanine Aminotransferase (ALT) was seen in our patient, they accompanied a parallel rise in CK, suggesting muscle to be the origin of those enzymes. We did not encounter cardiac and neurological complications. A case series on multiple wasp stings reported leukocytosis in 89% of their cases. Hence, leukocytosis is common in wasp stings and does not necessarily indicate infection.^9^ This was also apparent in our case, so antibiotics were only used judiciously.

Hemodialysis was carried out considering our patient's age and overall general condition. In addition, fluid therapy was restricted due to the development of respiratory fatigue due to increased respiratory efforts due to pleural effusion. Successful management of severe AKI with repeated hemodialysis, hemofiltration or peritoneal dialysis has been discussed in many reports.^11-13^ Since a kidney biopsy was not done, we could not confirm the exact mechanism of AKI in our patient. Since the CK levels were highly elevated, and there was no evidence of hemolysis or no prior episode of hypotension, our patient developed acute kidney injury probably secondary to rhabdomyolysis causing pigment nephropathy and acute tubular necrosis. Despite the course of the illness, our patient made a good recovery in the end. He was also satisfied with the treatment which he received.

Wasp stings remain an important occupational and environmental hazard in Nepal, where most exposures happen mainly between August and November.^3^ Sting-induced AKI, although described in the literature, is an uncommon complication. This condition has a good prognosis if treated with early supportive therapy and dialysis. Delay in care can lead to various complications and systemic manifestations. Physicians must have a sound knowledge regarding acute emergency care and further management of wasp stings, especially in rural areas of Nepal to prevent progression into serious complications. Cases of multiple wasp stings should be carefully monitored for possible complications and health care workers must be vigilant with a high degree of suspicion when dealing with such cases.
